# The worldly constituents of perceptual presence

**DOI:** 10.3389/fpsyg.2014.00450

**Published:** 2014-05-15

**Authors:** Ezequiel A. Di Paolo

**Affiliations:** ^1^Ikerbasque, Basque Science FoundationBilbao, Spain; ^2^Department of Logic and Philosophy of Science, University of the Basque CountrySan Sebastian, Spain; ^3^Centre for Computational Neuroscience and Robotics, University of SussexBrighton, UK

**Keywords:** predictive coding, sensorimotor contingencies, enactive cognitive science, counterfactual richness, perceptual presence, dynamical systems theory

In a thought-provoking article, Seth (2014) has elaborated a combined sensorimotor and predictive coding approach to perception. His proposal links perceptual presence with the counterfactual richness of predictive models in the brain, an appealing move, which is not without problems. Here I briefly state my main conceptual concern with the idea. I believe Seth can take this worry into account by shifting the emphasis from the brain to the worldly constituents of perceptual presence.

There are two broad ways of interpreting O'Regan and Noë's (2001) claim that perception depends on the mastery of the laws of sensorimotor contingencies (SMCs). They can be broadly depicted as conceiving mastery as *in-the-head* or *not-just-in-the-head*.

In-the-head notions of mastery can be sufficiently pinned down to states in an agent's functional architecture. Whether these are instantiated inside a skull or not, *pace* the label, doesn't matter; the resulting notion is internalist and representational. Not-just-in-the-head interpreters (e.g., Hutto and Myin, 2013) make appeals to non-representational forms of know-how to account for mastery.

Seth defends a marriage of predictive perception (PP) probabilistic models (an in-the-head approach, as he acknowledges) and SMCs theory (SMCT). This union could result in a powerful new version of SMCT, but it sounds as if one condition is for it to abandon its unwed enactive folly and move permanently into the household of PP and its in-the-head folks. Is this the inevitable option?

Refinements to PP allow Seth to explain both perceptual content and presence; the latter—this is the most interesting aspect—is associated with the counterfactual richness of predictive models. In a related move, Beaton (2013) has also argued that counterfactual reasoning is a natural partner for SMCT. And yet there remains something hard to pinpoint about this idea. What is the exact connection between veridicality and counterfactual richness? The matter is not so clear. Perceptual content and presence are not always so easily extricable. Witness Seth's own example comparing a free-standing ellipse with the same ellipse as part of a cylinder. A difference in counterfactual richness produces a perceptual difference in content! The ellipse now looks like a circle while presence seems unaffected; both lie equally veridical on the page. Content and presence are not independent (nor they need to be in Seth's account) and it seems that counterfactual richness can affect both.

The proposal does encourage an examination of synesthesia, a problematic case for SMCT. Seth proposes that different associationist theories could all be expressed in terms of intermediate level linkages in hierarchical generative models (HGMs) in the brain incorporating both the inducer and the concurrent in synesthetic experience. This link, he suggests, might be resistant to alterations by the prediction errors that arise as the inducer is encountered without concurrent stimuli, and that such resistance could be explained by an unusually high prior precision of intermediate level models where the association occurs.

Putting aside the question of whether this is not too strong an exception for the normal workings of HGMs—it may not be—why would intermediate models supersede low-level error-correcting mechanisms and so result in sustaining an impoverished repertoire of counterfactual probability distributions affecting concurrent presence (and not content) in synesthetes? It would be necessary to explain why, if both content and presence are expressed in probabilistic action-sensation mappings, and if counterfactual richness could affect both (ellipse-cylinder example), then error-correcting mechanisms at the lower level manage not to rectify for aberrant presence in synesthetes.

Seth's assumption, from what I understand, seems to be that there is no interference between how probabilities are encoded for actual processed stimuli and for counterfactuals.

Let us consider our first question again. What is the entailment between counterfactually-rich models and perceptual presence? Or more generally, what is the link between sensitivity, not so much to counterfactuals, but more specifically to the structure of action-dependent *virtual* likelihoods (in agent and world) and the veridicality of perception?

To answer this we must look at where counterfactual currency is cashed out, what is the source that informs counterfactual probability densities at all levels in the hierarchy. If we follow Seth's proposal eventually this must happen at the lower levels, at the interface with the world: counterfactual sensorimotor knowledge may then cascade through the hierarchy, but it must be informed by actual sensorimotor engagements. Here is why it is risky for a PP-based theory to assume that model attunement due to lower-level prediction errors could be prevented from propagating upwards in cases of synesthesia: the whole idea depends on maintaining some degree of accuracy and precision for counterfactual inferences too, i.e., it depends on regular updating by interactions with the world.

The necessary involvement of the world in determining presence shouldn't surprise us. It is here where not just actual events can get verified but the *real* structure of counterfactuals too. How? A lesson that can be derived from dynamical operationalizations of SMCs (Buhrmann et al., 2013), even if they have only been explored in very simple systems, is that there is a virtual (a nuanced sub-species of counterfactual) structure generated in the engagement between agent and world; more structure than just the *actual* sensorimotor trajectory. For instance, if I walk on a slope there is a strong downward tendency for my movement, even when I walk uphill. This is real and does not depend on my having enough sensitivity to detect it. Most “nearby” trajectory options are implied in the enacted movement. And the deeper or shallower grasp of this local dynamical landscape correlates with the more or less skillful enaction.

Perceptual presence could perhaps be *defined* as sensitivity to the virtual coherence that surrounds actual action, i.e., the real (actualized or not) aspects of the agent-world engagement. The more coherent they are found to be, the more present. This would be phenomenologically consistent with Seth's proposal.

Whether this sensitivity relies on counterfactually-rich HGMs, or alter-native—even non-representational(!)—mechanisms, the fact remains that it must also involve some worldly constituents, but not simply as inputs for the correction of modeling errors, because inputs are only actual (movements, sensory activations); witness, e.g., the striking difference in dynamical signature between a situated agent and one being fed a recorded input (Aguilera et al., 2013). The worldly constituents of presence play a role in shaping the dynamical possibilities of the agent-world coupling as in the slope example.

If this were not the case, then presence would be unrelated to worldliness and just in-the-head. If we eschew this possibility, we must also discard the sufficiency of counterfactual modeling richness for *fully determining* presence. This is not to say we must discard it as a factor *affecting* our sensitivity to virtuality if we were to continue exploring marriage arrangements between PP and SMCT; but on a more equal footing, as a theory of perception not-just-in-the-head.

## Conflict of interest statement

The author declares that the research was conducted in the absence of any commercial or financial relationships that could be construed as a potential conflict of interest.
